# Striatal Dopamine D_2/3_ Receptor Availability in Treatment Resistant Depression

**DOI:** 10.1371/journal.pone.0113612

**Published:** 2014-11-20

**Authors:** Bart P. de Kwaasteniet, Chedwa Pinto, Eric H. G. Ruhé, Guido A. van Wingen, Jan Booij, Damiaan Denys

**Affiliations:** 1 Department of Psychiatry, Academic Medical Center, Amsterdam, the Netherlands; 2 Brain Imaging Center, Academic Medical Center, Amsterdam, the Netherlands; 3 Department of Psychiatry, MC groep, Lelystad, the Netherlands; 4 Department of Nuclear Medicine, Academic Medical Center, Amsterdam, the Netherlands; 5 The Institute for Neuroscience, an institute of the Royal Netherlands Academy of Arts and Sciences, Amsterdam, the Netherlands; 6 University of Groningen, University Medical Center Groningen, Mood and Anxiety Disorders, Department of Psychiatry, Groningen, the Netherlands; National Institute of Health, United States of America

## Abstract

Several studies demonstrated improvement of depressive symptoms in treatment resistant depression (TRD) after administering dopamine agonists which suggest abnormal dopaminergic neurotransmission in TRD. However, the role of dopaminergic signaling through measurement of striatal dopamine D_2/3_ receptor (D2/3R) binding has not been investigated in TRD subjects. We used [^123^I]IBZM single photon emission computed tomography (SPECT) to investigate striatal D2/3R binding in TRD. We included 6 severe TRD patients, 11 severe TRD patients on antipsychotics (TRD AP group) and 15 matched healthy controls. Results showed no significant difference (p = 0.75) in striatal D2/3R availability was found between TRD patients and healthy controls. In the TRD AP group D2/3R availability was significantly decreased (reflecting occupancy of D2/3Rs by antipsychotics) relative to TRD patients and healthy controls (p<0.001) but there were no differences in clinical symptoms between TRD AP and TRD patients. This preliminary study therefore does not provide evidence for large differences in D2/3 availability in severe TRD patients and suggests this TRD subgroup is not characterized by altered dopaminergic transmission. Atypical antipsychotics appear to have no clinical benefit in severe TRD patients who remain depressed, despite their strong occupancy of D2/3Rs.

## Introduction

About one third of patients with major depressive disorder (MDD) do not respond to two or more trials with different classes of antidepressants and are considered treatment resistant [1], [2]. Treatment resistant depression (TRD) is associated with an overall worse prognosis and high medical costs [3]. At present, little is known about the pathophysiology of TRD, however several studies in TRD subjects demonstrated improvement of depressive symptoms after treatment with dopamine agonists [4]–[6]. These findings therefore suggest that abnormal dopaminergic neurotransmission is implicated in the pathophysiology of TRD [7].

In addition, aberrant dopaminergic neurotransmission is also associated with dysfunctional reward/motivational systems and anhedonia; the absolute or relative inability to experience pleasure. Anhedonia is one of the two key symptoms required for the diagnosis of MDD [8]. In TRD, anhedonia is often more profound and long-lasting and associated with a deficiency of the reward/motivational systems in the brain. Reward and motivation are mediated by the mesolimbic system, which is one of the major brain dopaminergic tracts [7]. This mesolimbic tract arises from the ventral tegmental area (VTA) and projects to the ventral striatum (including the nucleus accumbens), hippocampus and amygdala.

Relatively few neuroimaging studies examined the dopaminergic system in MDD with either positron emission tomography (PET) or single photon emission computed tomography (SPECT), and reported inconsistent findings [9], [10]. Studies investigating dopamine D_2/3_ receptor (D2/3R) availability reported increased striatal D2/3R availability in MDD patients compared to controls [11], [12], as well as increased striatal D2/3R availability in a subgroup of MDD patients with psychomotor retardation [13], [14]. Increased D2/3R availability may reflect either an up-regulation of D_2/3_ receptors, increased affinity of the receptor for the radioligand or a decreased synaptic dopamine concentration [7]. Therefore, the evidence of altered dopaminergic function in MDD is equivocal, also, as other studies demonstrated no differences between MDD and healthy controls [15], [16]. An explanation for these inconsistent findings may be that these studies included MDD patients with heterogeneous clinical characteristics which might underlie different clinical subgroups. Interestingly, it has been suggested that TRD is characterized by a more profound dysfunction of mood regulating networks relative to non-treatment resistant depression [17], [18], which suggests that TRD patients are at the worst end of a continuous depression spectrum. Furthermore, as TRD patients are often more severely anhedonic and psychomotorically retarded, and most of the time did not respond to serotonergic or noradrenergic drugs, abnormalities in TRD patients may be related to reduced dopaminergic signaling. To date, striatal D2/3R binding has not been investigated in TRD patients.

Therefore, the aim of the present study was to investigate striatal D2/3R binding in severe TRD patients to test the hypothesis whether TRD patients are characterized by diminished dopaminergic transmission, reflected by increased D2/3R binding. We performed in vivo measurements of striatal D2/3 binding in 6 TRD patients compared to 15 healthy controls. We additionally investigated the effect of antipsychotics on striatal D2/3R availability in 11 TRD patients and whether these drugs were associated with improvement of symptomatology.

## Methods

### Subjects

We included 6 TRD patients, 11 TRD patients on antipsychotics (TRD AP group) and 15 healthy control subjects matched for age and gender. TRD patients were recruited at the department of Psychiatry of the Academic Medical Center (AMC) in Amsterdam and St. Elisabeth Hospital in Tilburg. The study was approved by the Medical Ethical Committee of the AMC of the University of Amsterdam (METC AMC), and the Medical Ethical Committee of the St. Elizabeth Hospital (METC St. Elisabeth). All subjects provided written informed consent. Inclusion criteria for TRD and TRD AP subjects were: (i) age between 18 and 65 years; (ii) total Hamilton Depression Rating Scale (HAM-D) ≥18; (iii) primary diagnosis of MDD according to the Diagnostic and Statistical Manual of Mental Disorders (DSM-IV) criteria and assessed by The Structured Clinical Interview for DSM-IV (SCID) [19]. To capture the most severely TRD patients, we included only patients with an illness duration of >2 years, who did not respond to (i) at least two adequate treatments of two different modern antidepressants (selective serotonin reuptake inhibitors, serotonin–norepinephrine reuptake inhibitors, or noradrenergic and specific serotonergic antidepressants), and (ii) a tricyclic antidepressant, and (iii) an irreversible monoamine oxidase (MAO) inhibitor, and (iv) at least 6 sessions of bilateral electroconvulsive therapy (ECT). Exclusion criteria were: (i) Parkinson's disease, dementia or epilepsy; (ii) bipolar disorder; (iii) schizophrenia or a history of psychosis unrelated to MDD; (iv) alcohol or substance abuse during last 6 months; and (v) antisocial personality disorder. Healthy controls were screened by the structured clinical interview for DSM-IV disorders in order to confirm the absence of psychiatric or neurological illness [19]. None of the healthy participants reported a family history of psychiatric illness. We used the HAM-D [20] and Montgomery Asberg Depression Rating Scale (MADRS) [21] to quantify depression severity. The Maudsley Staging Method (MSM) was used to quantify the level of treatment resistance [22],[23]. The MSM score includes various clinical parameters; duration of the current depressive episode, symptom severity, and level of functioning as measured by the Global Assessment of Functioning (GAF) score. For a complete list of these clinical variables we refer to Fekadu et al [22].

### Single Photon Emission Computed Tomography protocol

SPECT scanning was performed using a 12-detector single-slice brain-dedicated scanner (Neurofocus, Inc., Medfield, MA, USA). Subjects underwent a measurement of the striatal D2/3R binding potential (BP_ND_) using the selective D2/3R antagonist [^123^I]iodobenzamide ([^123^I]IBZM). We applied a bolus/constant infusion technique, which has been described in detail previously [24], [25]. SPECT data were acquired for 60 minutes, starting 120 minutes after infusion of the radioligand. At the day of scanning subjects were not allowed to use alcohol, coffee and cigarettes since this has been associated with altered striatal dopamine release [26], [27].

### Image reconstruction and analysis

SPECT data were reconstructed in 3-D mode and attenuation correction of all images was performed as described earlier [28]. For quantification, a region of interest (ROI) analysis was performed. Fixed ROIs were positioned for the striatum and, as a reference, the occipital cortex [25]. Mean striatal and mean occipital binding were averaged from right and left ROIs. Then, BP_ND_ was calculated as the ratio of specific to non-specific binding ((total activity in striatum - activity in occipital cortex)/activity in the occipital cortex). All scans were analyzed by one investigator (CP) who was blind to the clinical data. To measure the inter-rater agreement, two authors (CP and BdK) independently analysed BP_ND_ in ten subjects. The intraclass correlation coefficient (ICC) was 0.94 for left- and 0.95 for right striatum which indicates an excellent agreement between both raters.

### Statistical analysis

Differences in age, HAM-D and MADRS scores were evaluated with a one way analysis of variance (ANOVA), and gender differences using a chi-square test. Comparison of striatal D2/3R availability between TRD, TRD AP and healthy control subjects was performed with an ANOVA as well. Using a Least Significant Difference (LSD) ANOVA post-hoc test, differences in D2/3R availability were investigated between TRD patients and healthy controls, between TRD AP patients and healthy controls and between TRD AP and TRD patients. Since D2/3R availability is influenced by age [29] and gender [30], we additionally included these variables as covariates in the group analyses using a one way analysis of covariance (ANCOVA). A two tailed probability value of 0.05 was selected as significance level.

## Results

### Patient characteristics

TRD, TRD AP and control subjects were comparable for age and gender (Table 1). HAM-D and MADRS scores did not differ between TRD and TRD AP patients which indicates no difference in severity of depression between both groups. Mean MSM scores of TRD patients were 11.8 (±1.0) and for TRD AP patients 11.8 (±0.5) which indicates a high level of treatment resistance in both groups. An overview of medication use of each TRD and TRD AP patient is reported in Table 2.

**Table 1:** Demographic and clinical measures of TRD, TRD AP and healthy control subjects.

Characteristic	TRD (n = 6)	TRD AP (n = 11)	HCs (n = 15)	p-value
**Age (years±SEM)**	48.7±3.7	55.9±2.0	54.5±2.0	0.17^1^
**Gender (female/male)**	3/3	7/4	10/5	0.63^2^
**HAM-D (SEM)**	20.2±1.3	22.2±1.5	n.a.	0.38
**MADRS (SEM)**	33.4±3.4	34.8±1.5	n.a.	0.66
**Duration of current episode in months (SEM)**	82±20.0	73±14.9	n.a.	0.73
**Age of onset (SEM)**	25.2±5.2	32.1±4.7	n.a.	0.36
**MSM scores (SEM)**	11.8±1.0	11.8±0.5	n.a.	0.98
**Psychomotor retardation Item 8 HAM-D (SEM)**	1±0.3	2±0.2	n.a.	0.02
**Striatal D2/3R availability (BP_ND_) (±SEM)**	0.84±0.06	0.50±0.06	0.81±0.05	<0.001^1^ TRD>HC: 0.75 TRD AP>HC: <0.001 TRD AP>TRD: 0.001

Abbreviations: TRD; Treatment resistant depression, TRD AP; Treatment resistant depression patients using antipsychotics, HCs; Healthy Controls, SEM; standard error of the mean MADRS; Montgomery Asberg Depression Rating Scale, HAM-D; Hamilton Depression Rating Scale, MSM; Maudsley Staging Method, BP_ND_; Binding Potential non-displaceable (reflects striatal D2/3R availability)

1 One way ANOVA.

2 Chi square test.

**Table 2:** Psychopharmacological drugs used in TRD and TRD AP patients.

Subject	TRD AP patients (n = 11)	TRD patients (n = 6)
**1.**	Olanzapine 5 mg Zoplicon 15 mg	None
**2.**	Lithiumcarbonate 600 mg Quetiapine 300 mg Lorazepam 3 mg	Tranylcypromine 10 mg Zopiclon 7.5 mg
**3.**	Quetiapine 500 mg Lorazepam 1 mg	None
**4.**	Tranylcypromine 90 mg Quetiapine 100 mg Aripiprazol 30 mg	Lithiumcarbonate 800 mg Zolpidem 20 mg
**5.**	Olanzapine 12.5 mg Flurazepam 15 mg Lorazepam 6 mg	None
**6.**	Quetiapine 600 mg Venlafaxine 75 mg	Mirtazapine 400 mg
**7.**	Dipiperon 80 mg Lorazepam 5.5 mg Zoplicon 7.5 mg	
**8.**	Quetiapine 700 mg Oxazepam 10 mg Zolpidem 20 mg	
**9.**	Tranylcypromine 20 mg Dipiperon 80 mg	
**10.**	Imipramine 25 mg Olanzapine 10 mg	
**11.**	Quetiapine 900 mg	

### SPECT imaging

**Figure 1:**
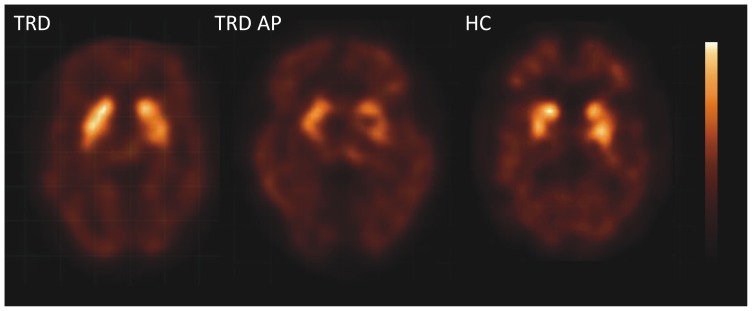
Transversal images of D2/3R availability. Transversal [123I]IBZM SPECT slices at the level of the striatum showing D2/3 receptor availability in a TRD patient, a TRD patient on antipsychotics (TRD AP), and a healthy control subject.

**Figure 2:**
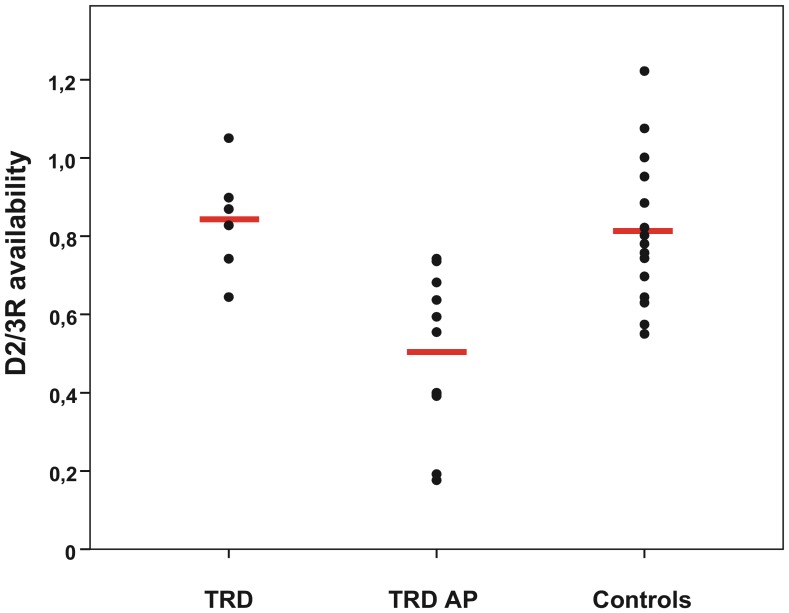
Striatal D2/3R availability for TRD, TRD AP and healthy control subjects. Striatal D2/3 receptor (D2/3R) availability of TRD patients, TRD patients with antipsychotics (TRD AP) and healthy control subjects. The black dots represents the striatal D2/3R availability of each subject. The horizontal lines indicate the mean D2/3R availability of each group which is 0.84 for the TRD, 0.50 for the TRD AP and 0.81 for the healthy control subjects.

There were no significant differences in mean striatal D2/3R availability between TRD patients and healthy controls (p = 0.75) suggesting that dopaminergic neurotransmission was not significantly altered in TRD patients (Table 1, Figure 1 and 2). The standardized effect size was 0.21. Furthermore, the mean D2/3R availability of the TRD AP group was significantly lower compared to both the TRD (p = 0.001) and healthy control group (p<0.001). Since the antipsychotics used by the TRD AP patients were all dopamine receptor antagonists, this demonstrates strong occupancy of striatal D2/3Rs (Table 1, Figure 2; occupancy of 50%±20%). Correction for age and gender did not significantly affect these results.

## Discussion

This preliminary study is, to the best of our knowledge, the first to investigate striatal D2/3R availability in TRD. We included a unique group of severe TRD patients which were eligible for deep brain stimulation, with an illness duration of more than 2 years defined as non-response to at least four adequate treatments of different antidepressants and at least 6 sessions of bilateral ECT. We showed no significant differences in striatal D2/3R availability in TRD patients relative to healthy controls which suggests that dopaminergic neurotransmission is not significantly altered in TRD. Furthermore, the TRD AP subjects showed significantly decreased striatal D2/3R availability relative to both TRD and healthy control subjects, which reflects a significant occupancy of D2/3Rs (estimated to be approximately 50%) by these atypical antipsychotics. Interestingly, despite these large differences in receptor occupancy depressive symptoms were not improved in the TRD AP subjects.

Previously, it was suggested that particularly TRD is associated with dopaminergic dysfunction [7]. Since TRD is characterized by a more profound dysfunction of mood regulating networks [17], [18], we expected them to show more severe dopaminergic dysfunction and as such an increased D2/3R availability compared to controls. Nevertheless, we observed no significant difference in striatal D2/3R availability in TRD patients compared to controls. We propose several explanations for this finding. First, other studies reported differences in D2/3R availability in psychomotor retarded patients [13], [14]. In our sample we used item 8 (range 0 to 4) of the HAM-D scores to measure psychomotor retardation which showed these TRD patients suffered only moderately from psychomotor retardation. Unfortunately, our study lacks more sensitive tests to measure motor retardation such as a finger tapping task [14]. We therefore cannot exclude the option that our patients were less psychomotorically retarded than in previous studies [13], [14]. Second, in the present sample TRD patients were only included after a non-response to MAO-inhibitors. As MAO-inhibitors increase dopamine concentrations, it could be hypothesized that especially in a subgroup of patients with a good response to MAO-inhibitors a hypodopaminergic state might exist. This could explain why in the current sample of non-responders to MAO-inhibitors no differences in striatal D2/3R availability were found. However, this hypothesis has not been investigated yet. Third, the present sample might be too small to detect differences in striatal D2/3R availability between TRD and control subjects. Importantly, however, the standardized effect size was small (d = 0.21). This implies that at least 343 patients should be included to demonstrate a significant group difference (at a statistical power of 0.8). Therefore the chance that future larger studies will find increased D2/3R availability in this subgroup of TRD-patients appears to be low. Furthermore, our present findings are consistent with several MDD studies which also reported no differences in striatal D2/3R availability relative to healthy controls [15], [16]. However these studies included different clinical groups with mostly treatment sensitive patients and a shorter duration of illness which therefore hampers direct comparisons.

As expected, the TRD AP subjects showed significantly decreased striatal D2/3R availability relative to TRD subjects (which reflects occupancy of D2/3Rs by the antipsychotics). The present D2/3R occupancy (approximately 50%) in the TRD AP group is comparable with that of atypical antipsychotics in schizophrenia patients [31], [32]. Since we showed no significant differences in depressive symptoms between these groups at adequate occupancy levels, this suggests that either monotherapy or augmentation with atypical antipsychotics does not provide clinical benefits in this specific TRD group, suggesting that these antipsychotics could be tapered in these patients. Importantly, all antipsychotic drugs used by the TRD AP patients have appreciable 5-HT_2A_ receptor occupancy which has been shown to improve depressive symptoms [33]. The 5-HT_2A_ receptor occupancy in these patients therefore cannot explain the lack of clinical improvement in this group. An explanation for the non-response might be that these atypical antipsychotics are all dopamine receptor antagonists. Interestingly, several studies showed that adjunctive dopamine agonists like pramipexole are effective in TRD patients [6], [34], [35] which suggests that dopamine agonist augmentation therapy might also be effective in the present severe TRD patients. We speculate that direct stimulation of dopamine D2/3 receptors may be helpful to increase motivational processes in the brain [36].

Despite the frequent use of atypical antipsychotic drugs in psychotic depression [37], [38], low-dose augmentation of these drugs in (non-psychotic) TRD patients has been proven to be effective [39], [40]. However, in these augmentation studies TRD was mostly defined as a non-response to only two trials of antidepressants. The present TRD patients additionally did not respond to more classes of antidepressants such as tricyclic antidepressants and MAO-inhibitors which may further explain the non-response to atypical antipsychotics, which might have no clinical benefit in more severe TRD patients. However, a randomized controlled trial would be necessary to definitely conclude whether antipsychotic augmentation in severe TRD is clinically useful.

We acknowledge several limitations of the present study. First, several studies showed that the striatum contains not only D2/3 receptors but also dopamine D1 receptors which operate via different intracellular pathways [41]. The dopamine D1 receptor is part of a D1-like subfamily which also comprises the dopamine D5 receptor [41]. Striatal D1 receptors are part of the direct nigrostriatal output pathway whereas D2 receptors are more prevalent in the indirect pathway [42]. Despite these functional differences, an animal study demonstrated that concurrent activation of D1 and D2 receptors in the shell of the nucleus accumbens produces a cooperative effect on the regulation of motivation, i.e. dopamine mediated reward processes [43]. Since depression has been associated with a dysfunctional reward/motivational system [44], [45], these findings suggest that altered expression of D1 receptors might lead to disturbances in the motivational system in MDD patients. However, as far as we know no human study has investigated striatal D1 availability in MDD nor in TRD. The Positron Emission Tomography (PET) radioligand [^11^C] SCH23390 binds to dopamine D1-like receptors [46], and to a lesser extent to D5 receptors. Since the expression of the D5 receptors in the striatum is lower, [^11^C] SCH23390 binding will predominantly reflect D1 receptor availability. [^11^C] SCH23390, but also other ligands like [^11^C]NNC 756 [47] or [^11^C]SKF 82957 [48] could therefore be used to investigate striatal dopamine D1 receptor availability in MDD and TRD patients.

Second, three out six TRD patients used psychotropic medication which might have influenced striatal D2/3R availability. One of these patients used a MAO-inhibitor which increases the synaptic dopamine concentration in the striatum [49]. Therefore, use of this drug could have reduced striatal D2/3R availability in this patient by increased competition with the radioligand. However, exclusion of this patient did not change results. In fact, large increases in dopamine concentrations are needed to reduce the [^123^I]IBZM binding in vivo. Another TRD patient used mirtazapine which is a noradrenergic and specific serotonergic antidepressant (NaSSA). Although mirtazapine has no affinity for dopamine receptors it does increase dopamine release in the prefrontal and occipital cortex by activation of the 5-HT_1A_ receptor and blockade of the α2-adrenergic receptors [50], [51]. However, there is no evidence that mirtazapine increases striatal dopamine release which suggests striatal D2/3R binding is not altered by mirtazapine use. Third, with [^123^I]IBZM we are able to measure striatal D2/3Rs in vivo. However, consequently we cannot exclude differences in extra-striatal D2/3Rs in TRD, which cannot be quantified. Finally, we did not select TRD-patients based on symptomatology like psychomotor retardation and/or anhedonia which might represent a subgroup with decreased D2/3R availability.

In conclusion, the present study did not detect differences in striatal D2/3R receptor availability in severely treatment resistant MDD patients relative to healthy controls. This contradicts the hypothesis that TRD is characterized by altered dopaminergic transmission. Furthermore, the results showed that additional treatment with antipsychotics decreased striatal D2/3R receptor availability (due to occupancy of D2/3R by the antipsychotics) in TRD. Importantly, because depressive symptoms were not reduced in these TRD AP patients, this suggest that in patients who have been administered different antidepressant drugs and remain depressed, atypical antipsychotics do not have a clinical advantage.
