# Correction: Striatal Dopamine D_2/3_ Receptor Availability in Treatment Resistant Depression

**DOI:** 10.1371/journal.pone.0135764

**Published:** 2015-08-13

**Authors:** Bart P. de Kwaasteniet, Chedwa Pinto, Henricus G. Ruhé, Guido A. van Wingen, Jan Booij, Damiaan Denys

The third author’s name is incorrect. The correct name is: Henricus G. Ruhé.

The correct citation is: de Kwaasteniet BP, Pinto C, Ruhé HG, van Wingen GA, Booij J, Denys D (2014) Striatal Dopamine D_2/3_ Receptor Availability in Treatment Resistant Depression. PLoS ONE 9(11): e113612. doi:10.1371/journal.pone.0113612.

The correct author contributions are: Conceived and designed the experiments: BDK CP JB HGR. Performed the experiments: BDK CP. Analyzed the data: BDK CP. Contributed reagents/materials/analysis tools: BDK CP JB. Wrote the paper: BDK CP JB HGR GVW DD.
